# Supplementary material for: MetaRibo-Seq measures translation in microbiomes
Source: Nat Commun. 2020 Jun 29;11:3268. doi: 10.1038/s41467-020-17081-z (PMC7324362; doi:10.1038/s41467-020-17081-z)
Supplement: Supplementary file 10 — Supplementary Data 7 [file 41467_2020_17081_MOESM10_ESM.zip › File2/Confidence_High_Taxonomy/155210_out.krona.html]

Javascript must be enabled to view this page.

members
magnitude
magnitudeUnassigned
count
unassigned
taxon
rank

155210\_out

159

superkingdom
159
2

976
159
phylum

159
200643
class

order
171549
159

family
171550
159

1

SRS146764\_contig\_number\_contig-100\_47527.47527
genus
239759
159

species

SRS024388\_contig\_number\_10000SRS043701\_contig\_number\_10643
214856
2

1
1946009

SRS893252\_contig\_number\_contig-100\_6245.6246
species

155
1118061

SRS011084\_contig\_number\_31605SRS011134\_contig\_number\_5027SRS011239\_contig\_number\_24084SRS011302\_contig\_number\_contig-100\_23557.107121SRS012969\_contig\_number\_12875SRS013098\_contig\_number\_3086SRS013158\_contig\_number\_contig-100\_4526.31966SRS013521\_contig\_number\_contig-100\_19484.19484SRS013800\_contig\_number\_contig-100\_8825.131781SRS014412\_contig\_number\_4170SRS014613\_contig\_number\_12668SRS014855\_contig\_number\_11368SRS015579\_contig\_number\_23799SRS015663\_contig\_number\_29739SRS015694\_contig\_number\_5639SRS015782\_contig\_number\_contig-100\_21704.156102SRS015794\_contig\_number\_4796SRS015854\_contig\_number\_15296SRS015960\_contig\_number\_14120SRS016095\_contig\_number\_4060SRS016203\_contig\_number\_6100SRS016335\_contig\_number\_20423SRS016954\_contig\_number\_18685SRS017103\_contig\_number\_2947SRS017191\_contig\_number\_11671SRS017521\_contig\_number\_contig-100\_5057.231370SRS017701\_contig\_number\_contig-100\_21553.57551SRS017821\_contig\_number\_12226SRS017916\_contig\_number\_6865SRS018351\_contig\_number\_14524SRS018427\_contig\_number\_9475SRS018541\_contig\_number\_11036SRS018575\_contig\_number\_contig-100\_12125.12125SRS018817\_contig\_number\_contig-100\_10065.85575SRS018836\_contig\_number\_21424SRS018936\_contig\_number\_9262SRS019381\_contig\_number\_13437SRS019445\_contig\_number\_2373SRS019496\_contig\_number\_12585SRS019685\_contig\_number\_29853SRS020271\_contig\_number\_contig-100\_3374.33758SRS020328\_contig\_number\_24037SRS020394\_contig\_number\_12924SRS021484\_contig\_number\_902SRS021948\_contig\_number\_contig-100\_7080.120583SRS022137\_contig\_number\_6385SRS022524\_contig\_number\_contig-100\_4188.90207SRS022609\_contig\_number\_contig-100\_26020.119163SRS023526\_contig\_number\_11296SRS023583\_contig\_number\_contig-100\_9749.86934SRS023971\_contig\_number\_contig-100\_847.44609SRS024625\_contig\_number\_16788SRS043768\_contig\_number\_28724SRS044535\_contig\_number\_contig-100\_8191.8192SRS045004\_contig\_number\_contig-100\_20309.114327SRS045645\_contig\_number\_18604SRS045713\_contig\_number\_contig-100\_19172.82581SRS045826\_contig\_number\_7465SRS046717\_contig\_number\_2658SRS048164\_contig\_number\_contig-100\_26773.111011SRS049402\_contig\_number\_15091SRS049773\_contig\_number\_10801SRS049896\_contig\_number\_20737SRS049959\_contig\_number\_29223SRS049995\_contig\_number\_18764SRS050925\_contig\_number\_4665SRS051031\_contig\_number\_31086SRS053335\_contig\_number\_contig-100\_13181.85544SRS053398\_contig\_number\_contig-100\_20980.100812SRS053649\_contig\_number\_1992SRS054352\_contig\_number\_12991SRS054905\_contig\_number\_17483SRS055017\_contig\_number\_34289SRS055982\_contig\_number\_6141SRS056273\_contig\_number\_13732SRS056519\_contig\_number\_contig-100\_5035.86098SRS057480\_contig\_number\_6231SRS063040\_contig\_number\_24344SRS064232\_contig\_number\_contig-100\_10692.10693SRS064276\_contig\_number\_18396SRS064557\_contig\_number\_12392SRS065504\_contig\_number\_7130SRS075078\_contig\_number\_3557SRS075773\_contig\_number\_22327SRS075821\_contig\_number\_19617SRS075878\_contig\_number\_216SRS075984\_contig\_number\_15211SRS076756\_contig\_number\_14722SRS077024\_contig\_number\_3531SRS077086\_contig\_number\_contig-100\_38879.38879SRS077127\_contig\_number\_5301SRS077194\_contig\_number\_29073SRS077231\_contig\_number\_5942SRS077294\_contig\_number\_18101SRS077502\_contig\_number\_16350SRS077552\_contig\_number\_6435SRS077641\_contig\_number\_contig-100\_29054.29055SRS077849\_contig\_number\_9669SRS078242\_contig\_number\_contig-100\_4380.159169SRS078419\_contig\_number\_12983SRS097958\_contig\_number\_17216SRS098061\_contig\_number\_9808SRS098571\_contig\_number\_15514SRS098644\_contig\_number\_31107SRS098655\_contig\_number\_2739SRS098717\_contig\_number\_12431SRS098881\_contig\_number\_17109SRS100907\_contig\_number\_contig-100\_4012.4013SRS101376\_contig\_number\_11251SRS101433\_contig\_number\_7937SRS103987\_contig\_number\_contig-100\_13041.13041SRS1041031\_contig\_number\_contig-100\_16589.49490SRS1041033\_contig\_number\_19251SRS1041118\_contig\_number\_contig-100\_1257.40388SRS1041132\_contig\_number\_3291SRS1041136\_contig\_number\_10381SRS1041139\_contig\_number\_contig-100\_2692.25298SRS104400\_contig\_number\_22265SRS104693\_contig\_number\_8874SRS104975\_contig\_number\_23253SRS105153\_contig\_number\_1117SRS1055022\_contig\_number\_contig-100\_18670.94676SRS140492\_contig\_number\_13195SRS140513\_contig\_number\_8291SRS142542\_contig\_number\_21921SRS142890\_contig\_number\_18773SRS142923\_contig\_number\_contig-100\_13857.96742SRS143372\_contig\_number\_243SRS143417\_contig\_number\_6692SRS143523\_contig\_number\_3654SRS143895\_contig\_number\_42050SRS143991\_contig\_number\_contig-100\_5143.136374SRS144135\_contig\_number\_35257SRS144183\_contig\_number\_1156SRS144297\_contig\_number\_11274SRS144362\_contig\_number\_22501SRS144506\_contig\_number\_46854SRS144537\_contig\_number\_19840SRS144714\_contig\_number\_contig-100\_6307.6308SRS145497\_contig\_number\_15459SRS146812\_contig\_number\_14092SRS147271\_contig\_number\_contig-100\_8067.198969SRS147346\_contig\_number\_29688SRS147425\_contig\_number\_11889SRS147766\_contig\_number\_contig-100\_18155.74444SRS148319\_contig\_number\_5830SRS148424\_contig\_number\_846SRS148721\_contig\_number\_32291SRS148817\_contig\_number\_10501SRS149075\_contig\_number\_819SRS149181\_contig\_number\_contig-100\_10833.130939SRS893230\_contig\_number\_contig-100\_8515.8516SRS893369\_contig\_number\_8994SRS893383\_contig\_number\_20231SRS971275\_contig\_number\_contig-100\_23651.134966
species
